# Endoscopy in the Diagnosis of Small Intestinal Tumors

**DOI:** 10.1155/DTE.3.153

**Published:** 1997

**Authors:** M. Kawaguchi, Y. Saitou, Y. Sakai, Y. Tani, S. Midorikawa, T. Sanji, Y. Handa, S. Morita, H. Ohno, H. Yosida, M. Turui, R. Misaka, T. Saitou

**Affiliations:** Fourth Department of Internal Medicine Tokyo Medical College 6-7-1 Nishishinjuku Shinjuku-ku Tokyo 160 Japan

## Abstract

The importance of endoscopy in the diagnosis of small intestinal tumors was evaluated in 15 patients with small intestinal tumors treated in our hospital. Two tumors were benign, and 13 were malignant (carcinoma in 5 patients, malignant lymphoma in 5 and leiomyosarcoma in 3). The presence of lesions could be determined by X-rays before surgery, but definitive diagnoses were difficult. When preoperative endoscopy of the small intestine was possible accurate preoperative diagnoses could be made based on the endoscopic findings and biopsies taken under direct vision. Endoscopy is therefore very important for the diagnosis of small intestinal tumors. It is necessary to develop small intestinal endoscopes that are easier to insert.


*Diagnostic and Therapeutic Endoscopy*, 1997, Vol. 3, pp. 153–160
Reprints available directly from the publisher
Photocopying permitted by license only

© 1997 OPA (Overseas Publishers Association)
Amsterdam B.V. Published in The Netherlands
by Harwood Academic Publishers
Printed in Singapore

### Endoscopy in the Diagnosis of Small Intestinal Tumors

M. KAWAGUCHI, Y. SAITOU, Y. SAKAI, Y. TANI, S. MIDORIKAWA, T. SANJI, Y. HANDA, S. MORITA, H. OHNO, H. YOSIDA, M. TURUI, R. MISAKA and T. SAITOU

*Fourth Department of Internal Medicine, Tokyo Medical College, 6-7-1 Nishishinjuku, Shinjuku-ku, Tokyo 160, Japan*

*(Received 17 January 1996; in final form 20 September 1996)*

**The importance of endoscopy in the diagnosis of small intestinal tumors was evaluated in 15 patients with small intestinal tumors treated in our hospital. Two tumors were benign, and 13 were malignant (carcinoma in 5 patients, malignant lymphoma in 5 and leiomyosarcoma in 3). The presence of lesions could be determined by X-rays before surgery, but definitive diagnoses were difficult. When preoperative endoscopy of the small intestine was possible accurate preoperative diagnoses could be made based on the endoscopic findings and biopsies taken under direct vision. Endoscopy is therefore very important for the diagnosis of small intestinal tumors. It is necessary to develop small intestinal endoscopes that are easier to insert.**

*Keywords:* Small intestinal tumor, small intestinal endoscope, video endoscope

### INTRODUCTION

Small intestinal tumors are encountered less frequently than gastric, large intestinal or esophageal tumors in daily medical practice. Small intestinal lesions are therefore looked for only after confirming the absence of lesions in the stomach, large intestine and esophagus. In addition, clinical symptoms of small intestinal tumors generally become manifest only at an advanced stage. Consequently, the prognosis of small intestinal tumors is usually poor when clinically diagnosed.

The present study, which focused on 15 patients with small intestinal tumors in whom thorough pathological examinations could be conducted, showed the importance of endoscopy of the small intestine in clinicopathological assessment and diagnosis of small intestinal tumors. Anatomically, the small intestine includes the duodenum, jejunum and ileum, but the duodenum is often excluded from statistical data and analysis on small intestinal tumors in Japan. In the present study, therefore, tumors in the jejunum and ileum were included but those in the duodenum were excluded.

### SUBJECTS

Fifteen patients with benign and malignant small intestinal tumors treated over the last 20 years period were included. One case of jejunal hamartoma was also presented, although it is not a true neoplasm.

### RESULTS

### Incidence by Type (Table I)

Of the 15 tumors, 2 were benign (1 jejunal leiomyoma and 1 jejunal neurinoma) and 13 malignant (5 malignant lymphomas, 5 carcinomas and 3 leiomyosarcomas).

### Age and Sex (Table I)

The average age of the 7 males and 8 females was 50. Both patients with benign tumors were female (mean age: 32 years). Of the 13 patients with malignant tumors, 7 were male and 6 were female (mean age: 52.8 years). The mean age and sex ratio (male:female) determined by type of disease were 53.6 years and 2:3 for malignant lymphoma, 54.8 years and 3:2 for carcinoma and 48 years and 2:1 for leiomyosarcoma.

### Incidence by Site (Table I)

Both benign tumors were located in the jejunum, while malignant tumors were in the jejunum and ileum in 8 and 5 patients, respectively. Malignant lymphomas were found in the ileum and jejunum in 4 and 1 patient, respectively, while carcinomas were located in the jejunum and ileum in 4 and 1 patient, respectively. Leiomyosarcoma was found in the jejunum in all 3 patients.

### Clinical Symptoms (Table I)

Presenting symptoms were anemia in 4 patients, hemorrhagic or tarry stools in 5, abdominal pain in 4, abdominal fullness due to ileus in 1 and intestinal perforation in 1. Symptoms due to hemorrhaging from tumors were thus most common, in 9 out of the 15 patients. The 2 patients with benign tumors also showed symptoms due to hemorrhaging. Of the 13 patients with malignant tumors, 7 had hemorrhaging, 4 had abdominal pain, 1 ileus and 1 perforation.

### Pre-operative Diagnosis and Diagnostic Methods (Table I)

Correct pre-operative diagnosis was made in 5 patients. The diagnosis was confirmed by endoscopy in all 5. A small intestinal endoscope was inserted from the mouth in 4 patients, while a colonoscope was inserted into the ileum through the ileocecal valve in the other. The presence of lesions was confirmed in 8 patients but qualitative diagnosis could not be made before surgery despite X-ray examination of the small intestine in 5, angiography in 2 and abdominal aortic RI in the other. Pre-operative diagnosis could not be made in 2 patients since they were operated on under an emergency basis under a diagnosis of acute abdomen. Recently, we have conducted endoscopy during surgery and reported findings in patients in whom the presence of lesions was confirmed but the diagnosis could not be confirmed before surgery.

### Prognosis (Table I)

Both patients with benign tumors are still alive. Four patients with malignant tumors died within 3 months of surgery, while 8 are still alive. The condition of the other patient with a malignant tumor is unknown. Of the 4 patients who died, 3 had malignant lymphomas in the ileum, while the other had jejunal carcinoma.

### CASE REPORTS

### A Case in Which Correct Diagnosis was Made before Surgery

A 28-year old male visited a nearby doctor when palpitations and tarry stools occurred in January 1992. Since his Hb was 7.8g/dl and occult fecal blood test were positive, upper GI tract endoscopy and barium enema study were conducted. He was referred to our hospital because the source of hemorrhaging could not be identified. A tumorous lesion was revealed by

Table I: Patients with small intestinal tumors (outcomes of intestinal malignancies)

| Case No. | Date | Age | Sex | Chief Complaint | Hb (g/dl) | Diagnosis | Preoperative Diagnosis | Method of Diagnosis | Location | Size of Tumor (mm) | Course after Operation |
|---|---|---|---|---|---|---|---|---|---|---|---|
| 1 | 1972 | 80 | F | Anemia | 12.1 | Carcinoma | Carcinoma | Endoscopy[2] | Jejunum | 45 x 15 | Unknown |
| 2 | 1977 | 54 | F | Abdominal pain | 9.7 | Carcinoma | Tumor[1] | X-ray[3] | Ileum | 34 x 28 | Alive |
| 3 | 1978 | 48 | M | Abdominal pain | 6.5 | Carcinoma | Tumor[1] | X-ray[3] | Jejunum | 56 x 44 | Died |
| 4 | 1980 | 47 | M | Anemia | 7.7 | Carcinoma | Carcinoma | Endoscopy[2] | Jejunum | 76 x 58 | Unknown |
| 5 | 1982 | 49 | M | Abdominal pain | 9.3 | Malignant lymphoma | Malignant lymphoma | Endoscopy[4] | Ileum | - | Died |
| 6 | 1986 | 74 | F | Ileus | 8.5 | Malignant lymphoma | - | Endoscopy[5] | Ileum | 160 x 53 | Died |
| 7 | 1990 | 62 | F | Abdominal pain | 9.3 | Leiomyosarcoma | Sarcoma | Endoscopy[2] | Jejunum | 35 x 28 | Alive |
| 8 | 1990 | 51 | F | Anemia | 8.1 | Malignant lymphoma | Tumor[1] | X-ray[3] | Ileum | 40 x 12 | Alive |
| 9 | 1990 | 22 | F | Perforation | 9.4 | Malignant lymphoma | - | Emergency surgery | Ileum | 50 x 38 | Died |
| 10 | 1990 | 48 | F | Melena | 4.7 | Neurinoma | - | MRI (Artery) | Jejunum | 60 x 55 | Alive |
| 11 | 1991 | 16 | F | Melena | 7.2 | Leiomynoma | Tumor[1] | Angiography | Jejunum | 55 x 48 | Alive |
| 12 | 1992 | 54 | M | Tarry stool | 8.3 | Leiomyosarcoma | Tumor[1] | Angiography | Jejunum | 80 x 45 | Alive |
| 13 | 1992 | 28 | M | Anemia | 7.8 | Leiomyosarcoma | Leiomysarcoma | Endoscopy[2] | Jejunum | 90 x 60 | Alive |
| 14 | 1992 | 72 | M | Tarry stool | 8.2 | Malignant lymphoma | Tumor[1] | X-ray[3] + Endoscopy[6] | Jejunum | 75 x 55 | Alive |
| 15 | 1994 | 45 | M | Melena | 6.8 | Carcinoma | Tumor[1] | X-ray[3] + Endoscopy[6] | Jejunum | 30 x 30 | Alive |

[1] Diagnosis upon discovery of the lesion.

[2] Endoscopy of small intestine.

[3] X-ray examination of small intestine.

[4] Colonofiberscopy.

[5] Endoscopic examination during emergency surgery.

[6] Endoscopic examination during surgery.

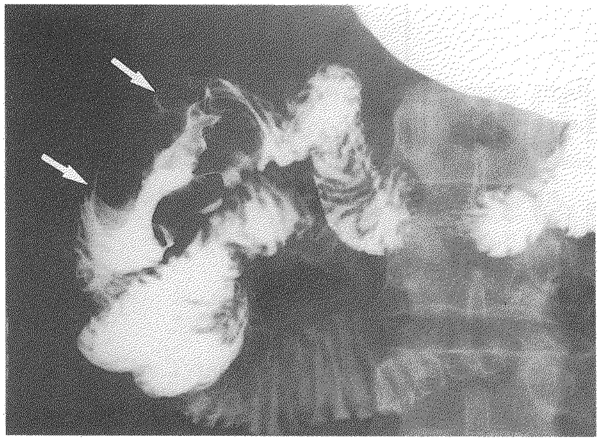

FIGURE 1 X-ray findings of the small intestine in case 13. A tumor with a gentle slope and a depression at the top is seen (arrows).

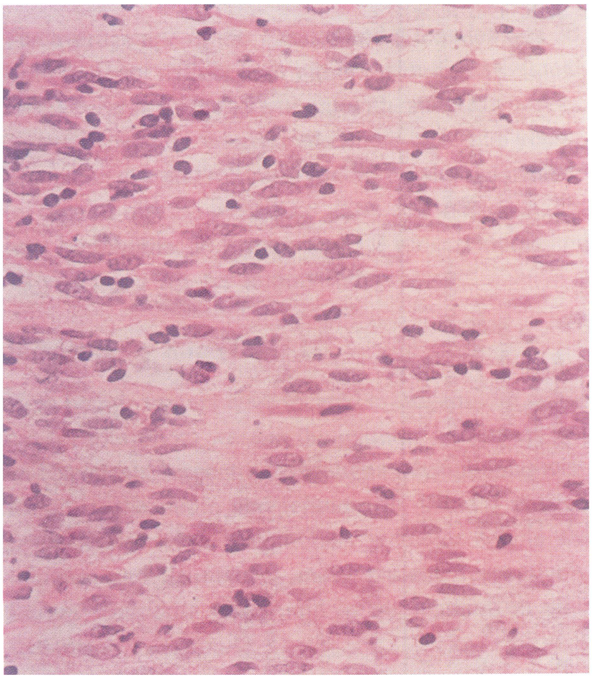

FIGURE 2 Endoscopic biopsy showed the tumor consist of spindle-shaped cells. A diagnosis of leiomyosarcoma was made.

roentgenography of the small intestine about 5 cm toward the anus from the duodenojejunal flexure. It had a gentle slope and was associated with a depression. The diagnosis of submucosal tumor was made (Fig. 1).

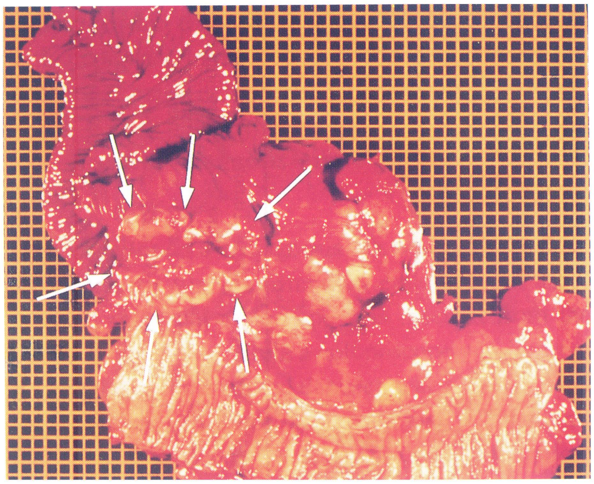

FIGURE 3 Macroscopic findings of case 13. The center of the tumor is collapsed and the surrounding margin is elevated (arrows).

Endoscopy of the small intestine also revealed a tumor with a depression at the site where X-rays of the small intestine had shown. The surface was smooth, and a diagnosis of submucosal tumors was made. A histopathological diagnosis of leiomyosarcoma was made from a biopsy of the margin of the depression (Fig. 2).

Partial jejunectomy and partial resection of the junction of the transverse and descending colons were carried out, and specimens obtained were macroscopically examined. The center of the tumor was collapsed. The bank-like elevation had a gentle slope (Fig. 3).

Histopathological examinations, as well as biopsy specimens, showed knotty patterns of spindle-shaped cells. Cell density was high, and mitosis was seen. The diagnosis of leiomyosarcoma was made based on these findings.

### A Case in Which Intraoperative Endoscopy was Useful

A 72-year old male had abdominal pain for about a year. He was kept under observation by a local doctor since upper GI tract X-rays and endoscopy, and barium enema study, revealed no abnormality. He was admitted to our hospital for detailed examinations because abdominal pain intensified. Upon admission, RBC was

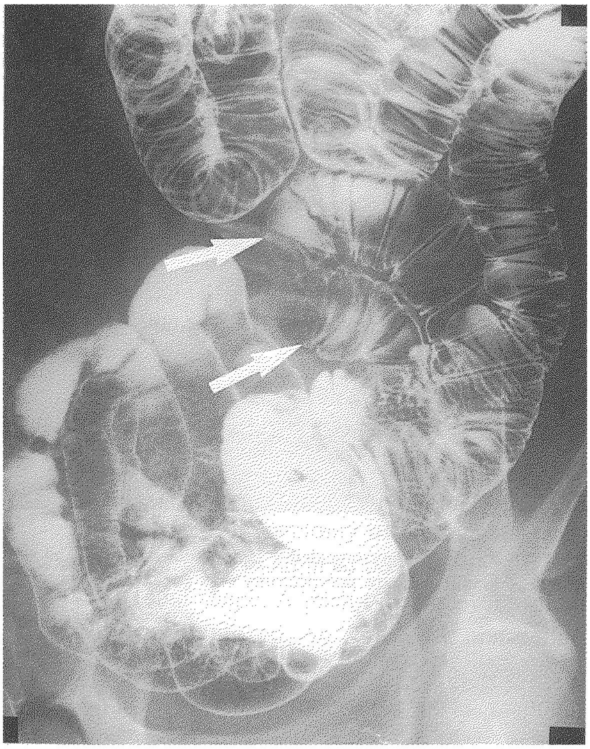

FIGURE 4A A tumor was discoverd in the jejunum in case 14.

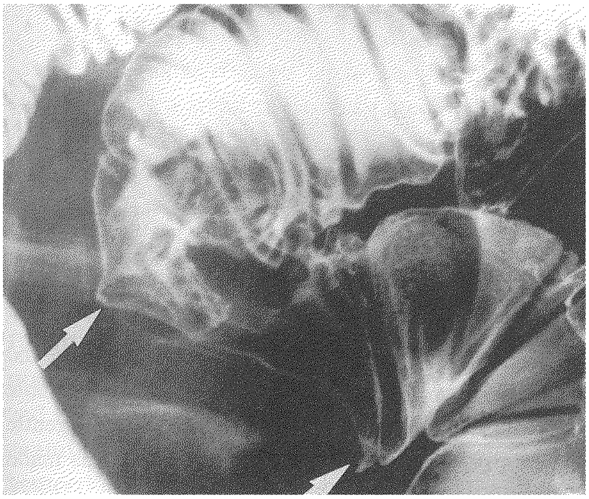

FIGURE 4B The slope is gentle and the surface is irregular (arrows).

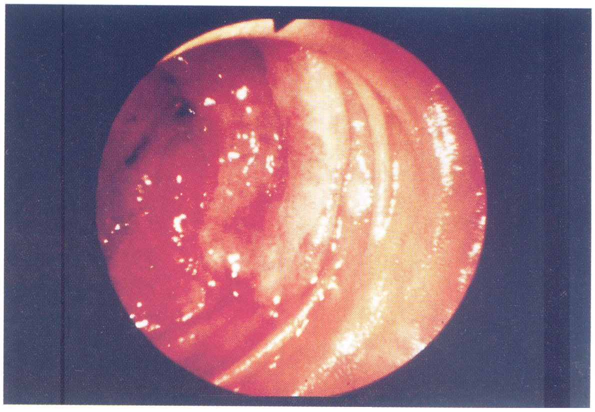

FIGURE 5 Intraoperative endoscopic findings of the small intestine in case 14 showed a large, plate-like friable elevation. The border between the tumor and non-tumorous mucosa is district.

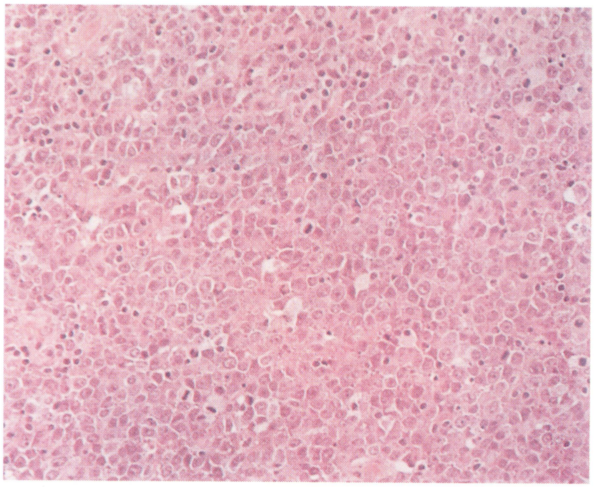

FIGURE 6 The tumor of case 14 consisted of large round cells with the large N/C ratio was large and prominent nucleoli. A diagnosis of non-Hodgkin's lymphoma of diffuse large cell type was made.

3,140,000 cells/ml, Hb 8.2g/dl and fecal occult blood reaction was positive. A tumor of about 6 cm in diameter was observed in the lower jejunum roentgenographically. The slope was gentle and the surface was irregular. A submucosal tumor, probably malignant lymphoma was suspected (Fig. 4).

Since small intestinal endoscopy before surgery was thought to be impossible because of the site, it was decided to conduct endoscopy during surgery. For this purpose, an endoscope for the upper GI tract was

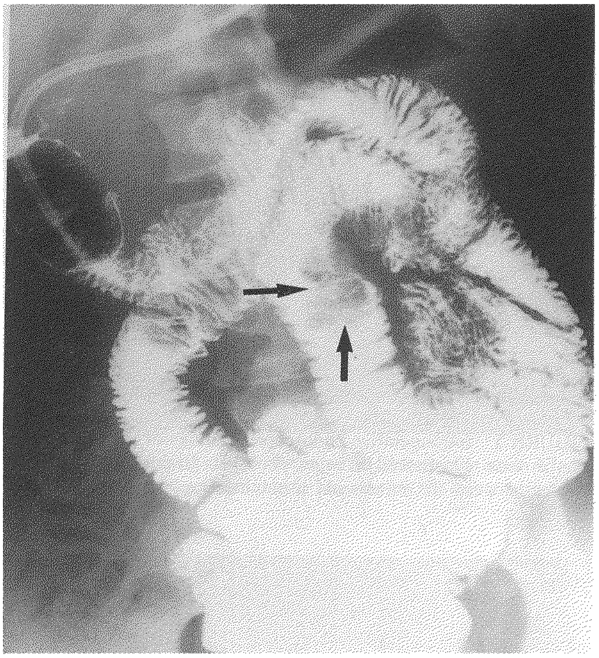

FIGURE 7A Roentgenography of the small intestine showed pedunculated polyp (arrows) 10 cm towards the anus from the duodenojejunal flexure.

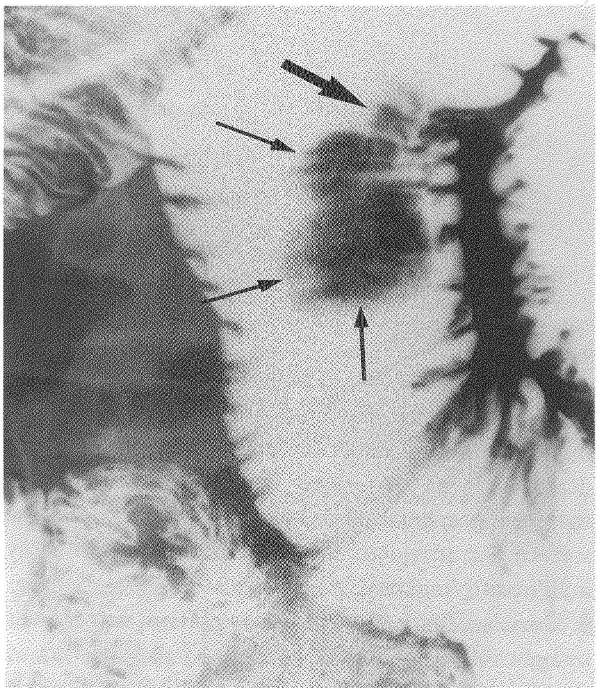

FIGURE 7B The polyp had a stalk (thick arrow) and the surface was rough and granular (thin arrows).

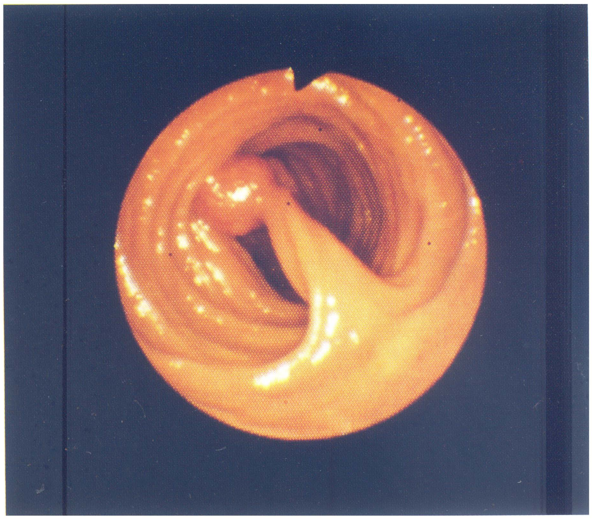

FIGURE 8 Endoscopy revealed a pedunculated polyp. The surface was granular. Snare polypectomy was performed.

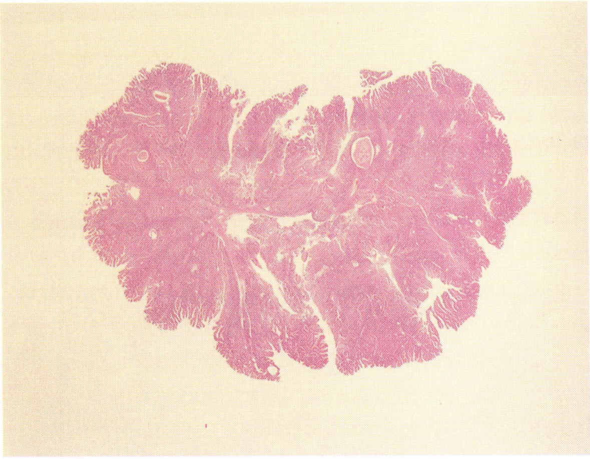

FIGURE 9 Histopathological findings of hamartomatous polyp. Muscle fibers show tree-like branching in the mucosa. The epithelium showed hyperplasia but not dysplasia.

inserted into the incision at a site 15 cm from the tumor. The tumor was a large flat plate-like friable elevation. The tumor margin had a relatively gentle slope. A diagnosis of malignant lymphoma was made based on the above findings (Fig. 5).

Histopathological examinations of resected small intestine specimens showed large round cells, large N/C

ratios and conspicuous nucleoli. A diagnosis of diffuse, large cell type non-Hodgkin's lymphoma was made (Fig. 6). Chemotherapy was initiated after surgery. The patient is being treated on an outpatient basis.

### A Case in Which Endoscopic Polypectomy was Performed

A 24-year old male complained of tarry stools (Hb 9.6 g/dl). Upper GI tract endoscopy was immediately carried out, but no abnormality was seen. Colonoscopy also revealed no abnormality. A pedunculated polyp of the small intestine 3.5 x 3.0 cm in size was recognized roentgenologically 10 cm from the duodenojejunal flexure. The surface was rough and granular (Fig. 7).

Observation and polypectomy using a small intestinal endoscope were thought to be possible, judging from the site and shape. A push-type small intestinal endoscope was employed. An elevated lesion with a peduncle was discovered about 10 cm from the duodenojejunal flexure. The surface was granular. Endoscopic polypectomy was carried out under a diagnosis of epithelial elevated lesion (Fig. 8).

Pathological examination showed tree-like branching of muscle fibers in the mucosae. The epithelium showed hyperplastic changes but no dysplasia. The diagnosis of hamartomatous polyp (Peutz-Jegher's type) was made (Fig. 9).

Small intestinal endoscopy repeated a week later showed no hemorrhaging from the polypectomized site, and only a small ulcer was noted.

### DISCUSSION

Tumorous lesions in the small intestines are often associated with hemorrhage, abdominal pain and ileus. Usually, the upper GI tract and large intestine are examined first to try to locate the site of hemorrhaging, and the small intestine is examined only when these examinations fail to identify any cause of the hemorrhage. Some investigators have found that angiography and 99m-Tc scintigraphy are useful for locating the site of the hemorrhage in the small intestine[1] but diagnosis by these methods is difficult in cases of active hemorrhage in which 0.5 ml/min or less blood is lost[2]. Roentgenography and endoscopy are therefore important for the diagnosis of tumorous lesions in the small intestine, as well as in other GI tract segments. In Japan, efforts to develop fiberscopes for the small intestine started around 1970[3], so experience in this field is still limited compared to endoscopy of other GI tracts segments.

At present push, ropeway and sonde types are available in Japan for endoscopy of the small intestine. From the beginning our unit has been involved in the development of push-type endoscopes[4]. At present, the Olympus model SIF-10 push type fiberscope is used in our unit for endoscopy of the small intestine (except during surgery when conventional endoscopes for the upper GI tract are used).

Endoscopy of the small intestine using push-type fiberscopes is conducted in the same manner as endoscopy of the upper GI tract segments except those procedures conducted under X-ray fluoroscopy to identify the insertion site. Push-type fiberscopes for the small intestine make it possible to examine the upper jejunum for about 50 cm toward the anus from the point of attachment of the ligament of Treitz in the duodenojejunal flexure.

Endoscopy is useful for the diagnosis of lesions in the small intestine. Small intestinal malignant tumors are often carcinoma, malignant lymphoma and leiomyosarcoma. Endoscopy and biopsy under direct vision are particularly important for differential diagnosis. Diagnosis by these methods is also important for cases of ulcerative lesions such as nonspecific small intestinal ulcers and Crohn's disease, as well as for erosive lesions such as protein losing gastroenteropathy and amyloidosis.

Whenever possible, we conduct endoscopy during surgery to confirm the site of the lesion and make a definitive diagnosis in patients in whom endoscopy of the small intestine cannot be conducted for various reasons before surgery.

We believe that, if possible, this procedure should be conducted whenever possible because it is also useful to determine the area that should be resected[5].

Since the push-type small intestine fiberscope is inserted over a long distance, close cooperation is essential between the operator who pushes the fiberscope and the one who adjusts its angle. Video endoscopes for the small intestine should be developed as soon as possible to facilitate this cooperation and make it possible to examine subtle mucosal changes more accurately.

Push-type fiberscopes enable examination of the small intestine to a distance 50–100 cm toward the anus from the duodenojejunal flexure, but deeper examination is difficult. Since this is possible with sonde-type fiberscopes[6], push-type and sonde-type fiberscopes should be combined as necessary to examine the entire small intestine.

### References

[1] Thompson, J.N. *et al.* Obsure gastrointestinal haemorrhage of small bowel origin. *Br. Med. J.* 1984; **288:** 1663–1665.
[2] Baum, S. *et al.* Angiography in the diagnosis of gastrointestinal bleeding. *Arch. Intern. Med.* 1967; **119:** 16–24.
[3] Tada, M. *et al.* Recent advances in enteroscopy. *Stomach and Intestine.* 1985; **20:** 723–731.
[4] Kawakami, M. *et al.* Fiberscopic diagnosis of small intestinal diseases. *Stomach and Intestine.* 1976; **11:** 167–174.
[5] Nomura, K. *et al.* A case of non-specific multiple small intestinal ulcers in which endoscopy during surgery was useful for making diagnosis. *J. of Surgery.* 1993; **55:** 913–915.
[6] Tada, M. *et al.* Radiologic and endoscopic diagnosis of the small intestinal tumors. *Clinical Gastroenterology.* 1995; **10:** 217–227.

